# Antibiotic use by poultry farmers in Kiambu County, Kenya: exploring practices and drivers of potential overuse

**DOI:** 10.1186/s13756-022-01202-y

**Published:** 2023-01-05

**Authors:** Jeniffer Waiyego Kariuki, Jan Jacobs, Marie Paule Ngogang, Olivia Howland

**Affiliations:** 1 Institute of Tropical Medicine, Antwerp, Belgium; 2 Department of Microbiology, Immunology and Transplantation, Leuven, Louvain, Belgium; 3 Faculty of Medicine, University of Yaoundé, Yaoundé, Cameroon; 4 International Livestock Research Institute, Nairobi, Kenya; 5 University of Liverpool, Liverpool, UK

**Keywords:** Antibiotic resistance, One health, Qualitative methods, Poultry, Drivers, Perceptions and practices

## Abstract

**Background:**

Antibiotic resistance is a global concern threatening achievements in health care since the discovery of antibiotics. In Kenya, this topic remains understudied in a context of rising demand for livestock products, intensification and the concomitant increase in antibiotic use. Our study investigates drivers and practices of antibiotic use in poultry farming. The study was conducted in Kiambu County, Kenya.

**Methods:**

A qualitative research methodology was employed: fourteen key informant interviews, twenty in-depth interviews, and four focus group discussions were undertaken. The interviews were semi-structured. Themes and subthemes from the interviews were generated through inductive analysis.

**Findings:**

Of the farmers interviewed, sixty eight percent were female, thirty three percent of the sampled farmers could not read, and the majority (eight five percent) of farmers had reared poultry for at least ten years. Research findings showed that farmers extensively used antibiotics. Antibiotic use was influenced by factors such as high disease burden, access to medicines and economic pressure. Common practices included prophylactic use, use of antibiotics to enhance production, self-prescription use, use of combination antibiotics (A combination antibiotic is one in which two or more antibiotics are added together for additional therapeutic effect.), and antibiotics classified as critically important in human medicine. Key information sources for the farmers were agro- veterinary dispensers, sellers of day-old chicks, and peer-learning. External factors driving the inappropriate use of antibiotics included access to the antibiotics, influence by marketers such as sellers of day-old chicks, and branding. Use of antibiotics was also driven by economic factors among the farmers, sellers of day-old chicks and agro-veterinary dispensers.

**Conclusions:**

Our findings indicate widespread use of antibiotics among poultry farmers in our study site. The use of antibiotics is influenced by an interplay of issues at the farmers’ level as well as broader social, economic and structural level factors. A multifaceted One Health approach focusing on regulatory frameworks, knowledge transfer, and research is required to promote stewardship and judicious use of antibiotics.

## Background

Antibiotics have significantly improved health by reducing morbidity and mortality from infectious diseases [1]. The benefits risk being eroded due to the continued emergence and spread of antibiotic-resistant bacteria [2]. The situation is especially dire as there is limited development of new antibiotics to replace those that have become less effective [2]. Antibiotic resistance increases patients' length of hospital stay, treatment cost, and morbidity and mortality. Estimates show that as of 2014, seventy thousand to two hundred thousand people die each year globally as a result of antibiotic-resistant related infections [2].

Low and Middle Income Countries (LMICS) are driving the increase in global consumption of antibiotics. Factors influencing this include high disease burden and unregulated access to antibiotics [3]. With increased antibiotic consumption, resistance in LMICs is widespread: multiple drug-resistant bacteria have been isolated from almost all countries on the African continent [4, 5].

Among the leading causes of the emergence of antibiotic resistance is the widespread use of antibiotics in livestock [6]. In LMICs, the use of antibiotics in livestock production is expected to increase by a 100-fold by 2030 due to increased demand for animal protein. Projections suggest that the demand for poultry meat in Nairobi, Kenya, will grow from six metric tons in the year 2000 to thirty thousand metric tons in 2030, with an accompanying 30-fold increase in production [7]. This increased poultry production, as observed elsewhere, is likely to lead to extensive use of antibiotics [8].

Most antibiotics used to treat infections in humans are also used in animals to enhance production or for the treatment of infection [9]. This creates an interdependence between human, animal and environmental health and a potential transfer of resistance. A One Health approach that entails the collaborative efforts of different sectors and disciplines is paramount to addressing this challenge. Describing the use of antimicrobials by farmers may provide valuable understandings of practices and possible drivers of antibiotic use and inform interventions to promote prudent use of antibiotics.

### Statement of the problem

Kenya has been reported to have a severe AMR^1^ problem: approximately two hundred different resistant genes have been identified in bacteria isolates. High prevalence of antibiotic-resistant bacteria in poultry has also been reported [10, 11]. Poultry farmers are reported to rarely seek consultation services from a veterinarian and therefore self-prescription of antibiotics is prevalent [12]. In this study, we suggest that poultry farmers have a significant role to play in how antibiotics are used. A recent study by Muloi et al. indicates that poultry farmers use critically important antibiotics such as colistin and fosfomycin in production [12]. There is, however, a dearth of information on how and why farmers use antibiotics in poultry farming and the possible drivers of antibiotic use. A better understanding of farmers’ practices and drivers of use may offer important insights into the drivers of antibiotic resistance in poultry production in Kenya. This, therefore is the main objective of our study.

## Materials and methods

### The study area

**Fig. 1:**
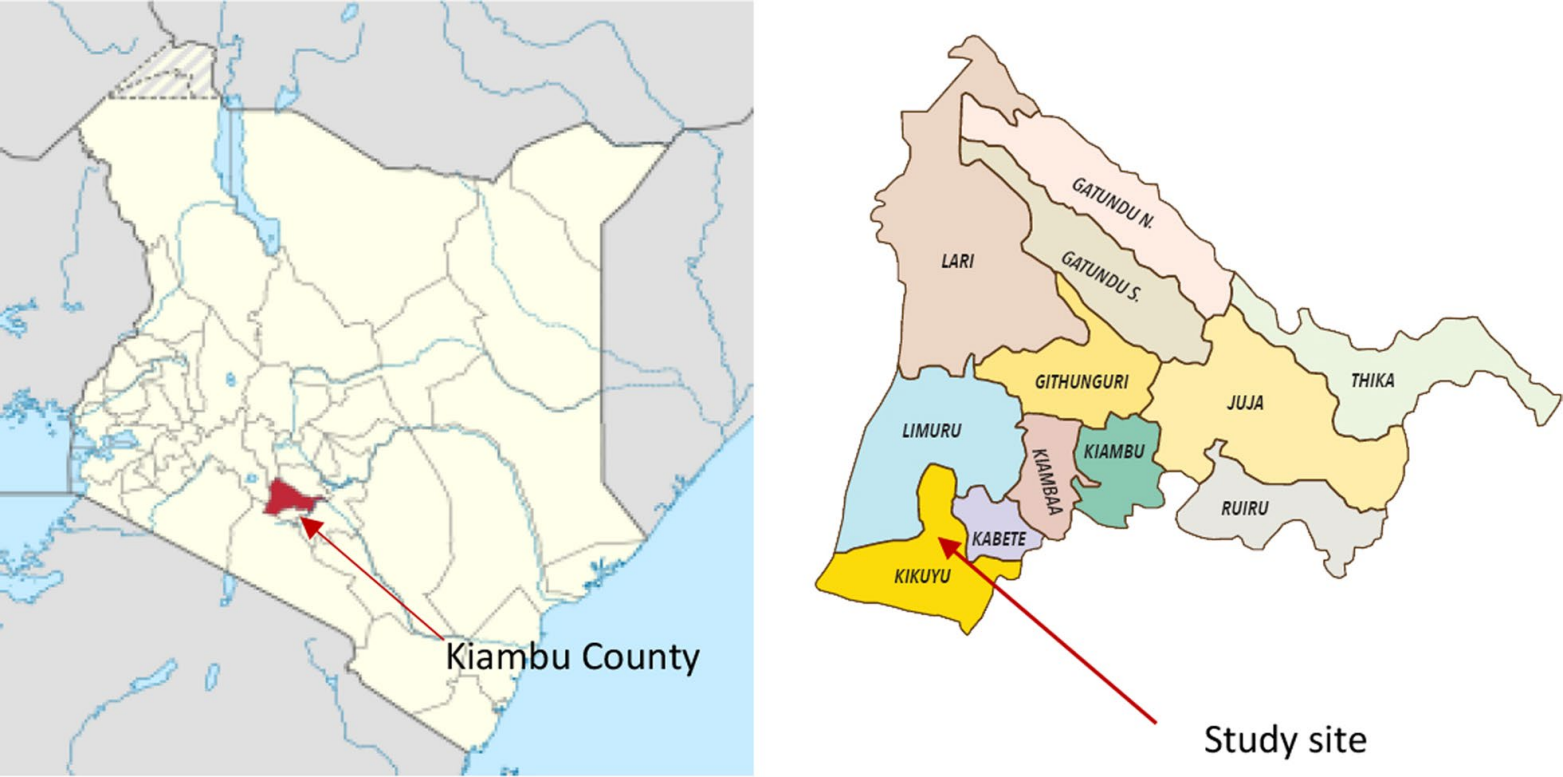
Maps of Kenya and Kiambu County showing the study site (Kabete Sub County) on the use of antibiotics by poultry farmers in their poultry farms (source: https://kiambu.go.ke/political-units/)

The study took place in Kiambu County in February 2019 (Fig. 1). Kiambu County is a peri-urban region in central Kenya. The County has an area of 2543.2 km^2^ and a population of 1,942,205 people [13]. The main economic activity of the population in Kiambu is smallholder farming, employing close to 75 percent of the population. Kiambu County produces poultry in large numbers, which may be due to the peri-urban location of the County [14]. According to the country-integrated plan 2018–2020, Kiambu County had a poultry population of approximately 2.5 million birds [13]. Kabete Sub County was selected because of the high chicken density and because it hosts the primary egg market in the County.

### Summary of study design and data collection methods

A qualitative study design was employed to shed light on the possible drivers of antibiotic use among poultry farmers in Kiambu County. The research team was composed of a veterinarian, an ethnographer and a professor of microbiology. Data for the study were collected through (1) key informant interviews, (2) focus group discussions (FGDs), (3) in-depth interviews with farmers, (4) observations and photographs. A semi-structured questionnaire with open-ended questions was used to guide the discussions and interviews.

### Description of study participants

The research was undertaken among poultry-rearing farmers. Poultry farms in Kiambu County can be classified as small, medium, and large-scale systems with the medium scale being the majority [15]. Small scale was classified as fifty birds or fewer, medium was classified as fifty to five hundred birds and large scale was between five hundred to six thousand birds. Key informants comprised three different groups. The first group included veterinarians and livestock production officers working in Kiambu County. These are trained personnel employed by the County Government for administrative purposes in livestock production, and have detailed knowledge of the livestock production system. The second group of key informants comprised agro-veterinary dispensers (AVDs) working in veterinary shops and animal feed shops in Kiambu County. They have diploma-level (college or post-high school vocational) education in animal health, and they are an important group as they serve the poultry farmers by dispensing veterinary medicine. The third group of key informants comprised chairpersons of poultry associations.

### Data collection

#### Key informant interviews

Fourteen key informants were interviewed using our semi-structured interview guide. Informants were selected through convenience sampling with the help of the local veterinarian. Criteria for selection included: professionals that had worked in agro-veterinary dispensaries, animal feed stores, as veterinarians or animal production officers for at least one year, and were engaged in this work full-time. Participants included two veterinarians, two livestock production officers, six AVDs, two livestock feed sellers, and two community-based leaders in the poultry industry (Table 1). Upon compiling the list of the professionals, we contacted them via telephone, explained the study, and booked appointments for an in-person interview at their convenience.

**Table 1:** Table showing a summary of participants interviewed on antibiotic use on poultry farms in Kiambu County

Method of data collection	Participant	Number of participants
Key informant interview	Agro-vet dispensers	6
	Field veterinarians	2
	Animal feed sellers	2
	Day old chick sellers	2
	Stakeholders in poultry industry	2
In-depth interviews	Poultry farmers	20
4 Focus group discussion	Poultry farmers	24

#### Focus group discussions

Four FGDs were undertaken. Each group had around six participants, with men and women represented. We chose to mix groups in order to ensure gender inclusivity. Group discussions lasted approximately one hour each. To ensure diversity in response, the study included participants from the three strata of poultry farming, *i.e.* small, medium, and large-scale farms. Farmers above eighteen years of age were selected and both male and female farmers were represented (refer to section "Participant profiles" for gender disaggregation). Two sampling methods were employed for the selecting the farmers who participated in the FGDs and interviews: purposive and snowball sampling. For the initial selection of participants, purposive sampling was used where farmers who fit within the above criteria were identified with the help of the local chief and division animal health assistant. For subsequent groups, snowballing was used where the first group of farmers (identified through purposive sampling) helped identify other farmers. The FGDs were undertaken in both Swahili and Gikuyu languages using a semi-structured guide containing open-ended questions. Data were recorded through notetaking and audio recording of the interviews. Information saturation was reached with the fourth group.

#### In-depth interviews

Twenty farmers were interviewed during the in-depth interviews. We conducted twenty interviews because this was the point at which saturation was reached. By saturation, we mean that a range of responses had been given and those responses were repeated with no new information. To triangulate^2^ information gathered through the focus group discussions and interviews, antibiotic use practices within the farm were also observed and photographs of products used within the farms were taken.

#### A note on gender and livestock

In the context of our study, male household heads traditionally make most financial decisions regarding the farm and livestock. Highly commercial ventures are controlled by men, and less commercial or smaller profit making ventures are the remit of women. Chickens are kept within the homestead and immediate area, and not taken out to pasture, and therefore are the responsibility of women. Livestock such as cattle or goats, which are also of high value, are the responsibility of men. This phenomenon has been reported elsewhere with both crops and livestock [16]. Broadly speaking, our study sample indicated that smaller-scale poultry farms were run by women and the larger ones (and therefore those which made larger financial profits) were run by men.

The first author is from this ethnic community, although not this geographical area and therefore has in-depth personal experience of cultural and social norms. It was important to us to ensure that a mix of genders was represented in the study, as well as making sure that farmers were selected based on their full authorization to make decisions on the farms.

### Participant profiles

Despite men traditionally being the heads of households, in our study the majority of farmers were female: sixty eight percent (30/44). About one-third (37 percent) were thirty to fourty years of age, eleven percent were forty to fifty years, while thirty three percent were fifty years and above. Most of the participants had a minimum of primary school education (66 percent). The majority (85 percent) of the farmers had been keeping poultry for more than ten years. However, we did not record gender on each individual transcript.

### Ethical clearance and informed consent

Ethical approval for the study was granted for one year through International Livestock Research Institute’s (ILRI) institutional research ethics committee Ref: ILRI-IREC2018-29 on 6/12/18.

Written consent was sought and gained from all participants prior to interviews or discussions.

### Data management and analysis

Data were anonymized and no names were connected with the written data. Basic demographic data were kept with each narrative such as gender and age of participant, as well as a general location and the type of farm. It was unnecessary for our records to keep personal information on participants. Data were stored on an external hard drive as a backup, which was kept in a secure location, as was the laptop computer used for data storage and management.

Data analysis was conducted using an inductive approach and thematic analysis. There was first a complete read-through of all the material collected from the key informants, FGDs and in-depth interviews. The data collected in Swahili and Gikuyu were translated into English by the first author. The data were then transcribed verbatim and imported into NVIVO 12 for data management and storage. We then analyzed the content of the narrative data and identified emerging themes and subthemes, and then organized the data within their relevant thematic categories.

## Results

### Practices around the use of antibiotics

#### Widespread self-prescribed use of antibiotics

Widespread, over-the-counter use of antibiotics was reported to be common among farmers. This was discussed by the farmers themselves, reported in FGDs, and from key informants. Key informants, such as extension officers, classified farmers’ use of antibiotics as ‘overuse’. This form of self-prescribing, participants said, was influenced by disease burden, knowledge of antibiotic brands, ease of access, and poor regulation of sale. Farmers in our study did not have access to regular veterinary advice, and there were no herd treatment plans in place. Farmers' reported that their choice of an antibiotic was informed by (1) previous successful use of the antibiotic for a similar condition, (2) popularity and availability of a brand, (3) broad-spectrum activity of the antibiotic, and (4) perceived potency of an antibiotic. The farmers in our study perceived the characteristics of the medicines through previous successful use, peer learning, and from the AVDs. The area was served by several agro-veterinary shops. Antibiotics were therefore easily available to the farmers, and farmers did not require a prescription to purchase them:

*There was a time when my birds were dying in large numbers, around 200 at a time, and I couldn’t treat them. I went to a doctor at Wangige who conducted the postmortem. He advised me to mix Limoxin and Tylodoxin. Following this, I discovered this combination to be very strong. So recently, I noticed that the birds were having some infections and I mixed the Limoxin and Tylodoxin again. If your birds are having diarrhea and respiratory disease, it is usually very severe, and they die immediately. Even then I make the mixture, I give birds for three days.*

Poultry farmer, Kiambaa.

#### Prophylactic and overuse of antibiotics for improved egg production

##### Use of antibiotics for disease prophylaxis

From FGDs and interviews with farmers, poultry diseases such as Newcastle disease, infectious bronchitis and omphalitis in day-old chicks were reportedly prevalent. Broiler and layer farmers reported using antibiotics as disease prophylaxis in day-old chicks and mature birds. Almost all farmers reported using antibiotics to protect chicks from disease upon arrival from hatcheries. The most commonly used antibiotic combination by market name for disease prophylaxis in chicks was product A (refer to Table 2 for expansion of brands). AVDs also ranked product A as the most commonly bought antibiotic combination for controlling disease in chicks. This brand was reportedly preferred because of its broad-spectrum activity. Farmers indicated that they used the antibiotics on the advice of sellers of day-old chicks for protection against infections. Both farmers and AVDs indicated that product A had gained popularity in the past year. For disease prevention in mature birds, sulphonamide-based antibiotics were reported by farmers in the FGDs to be the most popular.

To protect the day-old chicks from infections we use Product A which is an antibiotic for 7 days. We apply it in water; we also add glucose and liquid paraffin.

Poultry farmer, Gitaru.

Product A is mostly used by farmers for the day-old chicks. They prefer it because they say that it is powerful as it has a combination of four antibiotics and vitamins. We also like it a lot because it has broad-spectrum activity, you give it and you are sure.

AVD, Kikuyu.

**Table 2:** List showing commonly used antibiotic brand names, active ingredients and classification under WHO list of critically important medicines 2018

Product number	Trade name	Active ingredient	Drug group	WHO –critically important antimicrobials for human medicine
A	Aliseryl	Erythromycin	Macrolide	Critically important
		Oxytetracycline	Tetracycline	Highly important
		Streptomycin	Aminoglycoside	Critically important
		Colistin	Polymixin	Critically important
B	Tetracolivit	Tetracycline	Tetracycline	Highly important
		Colistin	Polymixin	Critically important
C	Fluquin oral solution	Enrofloxacin (not used in human but metabolized to ciprofloxacin)	Fluoroquinolone	Highly important
		Sulphamethoxazole	Sulfonamides	Highly important
D	Neoxy vitamin Ws	Neomycin	Aminoglycoside	Critically important
E		Oxytetracycline	Tetracycline	Highly important
F	Biotrim	Trimethoprim b.p	Sulfonamides	Highly important
		Sulphamethoxazole	Sulfonamides	Highly important
G	Trimovet	Trimethoprim	Sulfonamides	Highly important
		Sulphamethoxazole	Sulfonamides	Highly important
H	Tylodoxin	Doxycycline hydrate	Synthetic tetracycline	Highly important
		Tylosin tartrate	Macrolide	Critically important
I	Alamycin egg	Oxytetracycline	Tetracycline	Highly important
J	Limoxin	Oxytetracycline	Tetracycline	Highly important
K	Skajcycline	Oxytetracycline	Tetracycline	Highly important
L	Chick formula	Oxytetracycline HCl	Tetracycline	Highly important
M	Egcocin chick formula	Oxytetracycline	Tetracycline	Highly important
N	Esb3	Sulfaclozine sodium monohydrate	Sulfonamides	Highly important
O	Ampiclox	Ampicillin	Penicillin	Critically important
		Cloxacillin	Penicillin	Critically important

##### Use of antibiotics to enhance egg production

Participating farmers in the interviews and FGDs also discussed using antibiotics in sub-therapeutic doses as production enhancers. Products C, D and I (tetracycline) were the most popular, marketed to enhance egg production of layers. Equally, AVDs reported that these products sold relatively quickly. Farmers said that they associate the containers' yellow color and a picture of an egg with increased production. Commonly, antibiotics were administered from a young age until the start of the laying period. Antibiotics were also administered whenever farmers noticed that birds had production problems. On individual farm visits, product I containers were the most common, suggesting that this product was commonly administered compared to others.

For the Alamycin egg, I use this when the birds are young. I start giving the birds when they are about one month old and continue until they start laying. I also give when the production goes down until they start laying properly.

Poultry farmer, Ndumbuini.

**Fig. 2:**
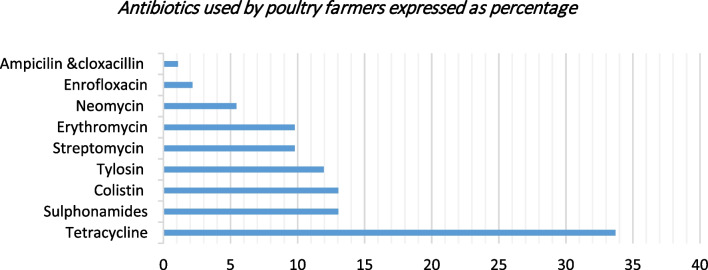
Showing the antibiotics commonly used by poultry farmers in their farms in Kiambu expressed as a proportion of the farms using antibiotics in the past six months (n = 44)

Of the farmers interviewed, 78 percent (34/44) reported the use of an antibiotic in the past six months prior to the study (refer to Fig. 2). Most of the farms where antibiotics were used were large scale where broilers and layers were reared. On the farms where antibiotics were used, 47 percent used combination antibiotics.

##### Support of diagnosis by laboratories

AVDs reported mainly depending on symptomatic disease diagnosis. In exceptional cases such as high mortality rates, farmers were referred to the government and university laboratories located near most farms. Though farmers in FGDs indicated laboratory charges were affordable, seeking diagnostic services was constrained by a long turnaround time for processing of results.

Yes, we take our chickens to the Kabete laboratory especially when they are dying in large numbers so that they can determine if the problem originates from the hatchery. The only problem is that it takes a long time. Sometimes I just choose to go to the agro-veterinary shop although the prices are not high at the laboratory. There is laxity on the government side, they work slowly and sometimes you may hear that they have gone on strike.

Poultry farmer, Kanyariri.

### Drivers of antibiotics use

#### Economic drivers of overuse of antibiotics

##### Economic influence on disease control

In the FGDs profit maximization was reported to be a significant driver influencing disease control practices for the farmers in our study, and antibiotic compliance practices such as the observation of a withdrawal period of antibiotics were not always observed. All interviewed farmers indicated that they operated on very tight profit margins. Prices of eggs had dropped from approximately USD 3.00 per tray to less than USD 2.00 per tray (a tray has thirty eggs). However, animal feed cost was reported to be quite high compared to profit margins realized from sale of either eggs or meat.

*Poultry rearing has also become very unprofitable because of the bad markets; therefore, our profits margins are very little.*

*Poultry farmer, Kinoo.*

This is important because if poultry are not financially productive because they are sick, then farmers will feel the need to administer antibiotics. Cost of operation played an important role in disease control, significantly constraining health seeking behavior of poultry farmers for their chickens. Farmers perceived the cost of on-farm veterinary services to be high, yet on the other hand, consultation at the agro-veterinary shop was offered as a free service as the farmer was expected to buy medicine after consultation. Field animal health professionals also indicated that farmers were hesitant to have farm-level consultations, as the fees charged for this service were relatively high.

*Most farmers do not like calling the veterinarian because they think that they know and therefore they go and ask for the medicines directly. They also do not like paying for the consultation services, whether you treat the birds or not. Here you have to do something as the doctor so that they can pay. Most farmers are small-scale farmers and so they do not expect you to charge anything. Sometimes they will ask you; did you make all this money in the short period that you were here. Sometimes they will think for instance how comes I charged USD 50.00 in such a short period that I was with them.*

*Field veterinarian, Kanyariri.*

From the FGDs and interviews with the farmers, the cost of routine vaccinations was perceived as prohibitive. Even though the farmers were aware that vaccination should be undertaken regularly, the cost of vaccines was cited as a limiting factor. Most vaccines were packed in 100 doses and required refrigeration, yet indigenous poultry farmers owned an average of 20 chickens. The majority of these farmers considered it uneconomical to purchase 100 doses if only 20 birds needed to be vaccinated. Cost also limited the observation of important bio-security measures such as frequent changing of bedding in the chicken houses.

*For the indigenous birds, we also use some antibiotics but not as much as what is used in the large scale poultry farming. On vaccination of the indigenous birds we do not often vaccinate them because the vaccines are packed in large doses and therefore it is not economical to vaccinate. *

*Poultry farmer, Uthiru.*

#### Economic influence on dispensers of antibiotics

Economic motivation was discussed by the AVDs and key informants to contribute to the overuse of antibiotics. As the consultation services offered by the AVDs were free, the farmers had to purchase some medicines from the agro-veterinary for the enterprises to remain viable. Market competition among the agro-veterinary shops was also reported by the AVDs and field veterinarians to be a driver of the use of more potent antibiotics perceived by the veterinarians and dispensers. The AVDs were compelled by competition to give potent antibiotics to ensure positive outcomes on disease control and customer retention.

*Some agro-veterinary shops also prescribe strong [more potent] antibiotics so that they can create a good name for their shops because of competition.*

*Field Veterinarian.*

*You have to be very careful because if the bird does not respond to medicine they may not come to your shop again as there are nine more agro-veterinary shops around here.*

*AVD in Wangige.*

## Discussion

Our study contributes to the existing understanding of antibiotic use by poultry farmers in their farms. It demonstrates that farmers’ use of antibiotics is driven by an interplay of social and economic factors. Injudicious use enhances the risk of AMR in animals, humans and the environment.

### Infection control practices

Diseases posed a significant challenge among the interviewed poultry farmers driving antibiotic use. Central to reducing antibiotics use is disease control through measures such as biosecurity procedures and vaccination. This has been demonstrated among pig farms in Belgium, where biosecurity level was associated with the amount of antibiotics used [17]. Biosecurity in poultry production is anchored on three core principles: cleaning, segregation, and disinfection [18]. Combined, these measures reduce the risk of introduction and spread of disease. In our study, there was a low level of adoption of biosecurity measures. Cost–benefit analysis of biosecurity may act as an incentive, encouraging farmers to implement these measures in their poultry farms [18].

In commercial chickens, vaccination coupled with the use of biosecurity measures may significantly reduce antibiotic use without compromising levels of production [19]. In our study, most broiler and layer farmers vaccinated their birds against infectious poultry diseases, although not routinely. A study of beef farmers in Tennessee in the USA found that packing vaccines in large amounts was a key hindrance in the purchase and use of vaccines, as was similarly reported by keepers of indigenous birds in our study [20]. To encourage the utilization of vaccines by indigenous bird keepers, manufacturers should consider packing vaccines in smaller numbers of doses.

### Use of antibiotics for disease control and production

Similar to other studies conducted among poultry farmers in LMICs [21, 22], we found that antibiotics were reported to be used in sub-therapeutic doses to enhance production, especially in layers and broilers. The application of antibiotics in sub-therapeutic doses results in selection pressure stimulating the emergence of resistant bacteria [23]. Similarly, studies in Vietnam and Cambodia found that antibiotics were widely used to protect day-old chicks against infections on arrival [24, 25]. The study in Vietnam does not explain the drivers of this practice, but findings of our study strongly point to the influence of sellers of chicks encouraging antibiotic usage for the prevention of omphalitis. Poor sanitary conditions at the hatchery and on the farm are linked to a high prevalence of omphalitis in chicks [26]. The use of antibiotics for prophylaxis may affect the curative use of these antibiotics in human and animal medicine.

In our study, the most commonly used brand for prophylaxis in day-old chicks*,* product A (refer to Table 2 for reference on active ingredients), gained popularity in the past year. There may have been an economic motivation for the sellers of the chicks to market this product. To effectively reduce the use of antibiotics in poultry, suppliers of chicks form a very important target group.

### Use of combined antibiotic brands and critically important antibiotics in poultry production

In our study, a significant proportion of antibiotic brands used (53.3 percent) contained more than two different groups of antibiotics sold as a single product. For instance, product A, the most commonly used antibiotic brand for disease prophylaxis in chicks, had a combination of four different important antibiotics (ref to Table 2). The use of combination antibiotics has been reported as a key driver of the emergence of multiple drug-resistant bacteria due to the exposure of bacteria to different antibiotic classes [27].

The World Health Organization (WHO) lists the majority of antibiotics used for prophylaxis of disease in our study as important and critical medicine in human health. In our study, colistin use was reported in 13 percent of poultry farms. This is consistent with the findings of Muloi et al., who reported that colistin was an antibiotic of choice for poultry farmers: 16 percent of veterinary shops dispensed colistin to poultry farmers in Nairobi, Kenya [12]. The study by Muloi does not explain the drivers of this practice. As previously indicated, our findings point to the promotion of the use of this antibiotic by sellers of day-old chicks with a view to prevent omphalitis. While the use of colistin in poultry production has been banned in countries such as China because of its human medical importance, in most LMICs it is still used in livestock production [4, 21, 28, 29]. The widespread use of colistin in livestock production in China is thought to be a significant driver in the emergence of plasmid-mediated MCR-1 in *Enterobacteriales* isolated in humans [30].

In our study, the use of poultry droppings as animal feed was very common, and poultry was often housed with other species such as cattle and pigs. This creates potential pathways for the transfer of antibiotic-resistant bacteria including transfer to the environment, to cattle and pigs fed on the droppings, and ultimately to humans at the top of the food chain. Colistin is excreted in its bioactive form. Therefore, the antibiotic is available in sub-therapeutic doses in chicken droppings and may induce selection pressure in the gut of the animals that consume the poultry droppings as animal feeds. This creates an avenue for the spillover of resistant microbes from animals to humans through physical contact or the food chain. Similar strains of resistant genes have been reported in humans and animals, and examples include plasmid-mediated resistance to colistin in *Klebsiella* spp, suggesting transmission of AMR from animals to humans [31, 32]. This underscores the need for a One Health approach through multiple sectoral and cross-disciplinary cooperation to address the AMR challenge.

### Source of veterinary services

Farmers sought veterinary services from AVDs but as noted in a study from Ghana, they did not form part of the farm management [21]. In our study, the high cost of on-farm consultation was reported to be a key hindrance to the involvement of veterinary professionals in the management of poultry farms. In addition, while there were veterinary laboratories near farms, most farmers and AVDs did not utilize them citing the long turnaround time for results. Lack of integration of prescriptions with extension services or laboratory diagnosis results in the use of broad-spectrum antibiotics for perceived improved treatment outcomes. Although in many contexts, drugs are administered without laboratory diagnoses, in the context of our study, very few farmers ever sought such services.

Self-prescription of antibiotics was the second most important route to seeking veterinary services for poultry farmers. This was driven by widespread knowledge of antibiotic brands compounded by factors such as ease of access to antibiotics and financial pressure (the high cost of veterinary consultation). Antibiotic resistance is reported to be higher in settings where self-prescribed antibiotics are used frequently [33, 34]. In LMICs, where the sale of antibiotics is poorly regulated, delinking financial gains from the sale of antibiotics has been suggested as a possible intervention toward reduction of self-prescription use of antibiotics [34].

### Recommendations

To increase the efficacy of farmers’ understanding of AMR, research programs should adopt a collaborative effort between social scientists, environmental scientists, animal health and human health practitioners, *i.e.* a One Health approach. This will give a more complete picture of the risks we are facing from possible overuse of antibiotics, and a better understanding of farmers’ needs. Actions such as surveillance of antimicrobial use and resistance when implemented in synergy across disciplines and sectors increase the potential for the reduction of AMR.

Probably the most salient factor in antibiotic use is financial. If farmers could be shown more effective and either free or inexpensive ways of keeping their poultry healthy, they would be less likely to overuse antibiotics. Day-old chicks are very vulnerable to disease and so farmers are immediately dosing them with antibiotics. One important node for any future intervention would be supplier of these chicks to ensure that they are healthy when they reach the farmer.

## Conclusions

Our findings indicate widespread use of antibiotics among poultry farmers in our study site. The qualitative methodology provides in-depth insight into some of the drivers of the regular use of antibiotics by poultry farmers, which would likely not have been revealed through quantitative methods. Although specific to this geographical location, our findings contribute to a broader body of evidence on antibiotic use in poultry across LMICs. In our study, findings indicate that the use of antibiotics is influenced by an interplay of social and economic factors at the farmers’ level as well as broader social, economic and structural^3^ level conditions.

## Data Availability

Qualitative data for this study may be made available upon request from the corresponding author.

## Appendix

**Table:**

Theme	Sub-theme	Questions
Semi structured questionaire		
Practice	Self-prescription and medication Peer learning(leaning from other farmers	When and why did you start rearing poultry? Do you rear commercially or as subsistence? What is your market for your poultry—eggs? Meat? Very local or further afield markets? What are some of the challenges that you face while rearing your poultry? How do you keep your poultry healthy and productive? When your chickens get sick what do you normally do? Who advises you on how to take care of your chickens? On which occasions do you call a veterinarian? Do you use any traditional medicines for your chickens? What are some of the medicines that you use in the production of your chickens? Who purchases and who administers medicine when chickens get sick? How do you know the amount to use?
Knowledge	Literacy Education on antibiotic use by dispensers Knowledge on disease control Identification of antibiotics Identification antibiotics by trade names Knowledge on antibiotic resistance Source of information for antibiotic use’	What is the level of education for the poultry farmers? Are you able to read and understand the information provided with the medicines? Does the dispenser provide information to you when they sell the drug? If so which information? Are you aware of antibiotic resistance? Please explain How does anti-biotic resistance develop? What are the consequences of antibiotic resistance? Who is at risk from antibiotic resistance Can using antibiotics in poultry have effect in humans, if so what are some of these effects? What is withdrawal period and why is it important?
Attitude	Expectations Disease control Tradition beliefs Possible benefits of antibiotic use on the poultry Risk perception of antibiotic use Cost issues (veterinary consultation, antibiotics)	Are there any expectations that you have of veterinarian when treating your chickens? What are the biggest issues for you in the health of your chickens? What are the biggest issues you see in accessing medicines or medical knowledge for your chickens? What is your opinion on the use of antibiotics /the impact of use on the chickens? To what extent do you think use of antibiotic can be dangerous to humans and animals

## Abbreviations

AMR: Antimicrobial resistance
AVDs: Agro-veterinary dispensers
FGDs: Focus group discussions
LMICs: Low and middle-income countries
MCR: Mobilized colistin resistance
WHO: World Health Organization

## Footnotes

1 Antimicrobial resistance.

2 By which we mean collection of the same information using more than one method, which aids in gaining an in-depth and rigorous understanding of a phenomenon.

3 By which we mean issues at the level of government policy, as well as national and international political conditions.
